# An Incidentaloma: Primitive Neuroectodermal Tumor of the Thymus

**DOI:** 10.1155/2011/407523

**Published:** 2011-09-22

**Authors:** Suzanne M. Smith, Abigail Berniker, Stephen B. Iorfido

**Affiliations:** ^1^Department of Internal Medicine, Mercy Catholic Medical Center, Mercy Fitzgerald Hospital, 1500 Lansdowne Avenue, Darby, PA 19023, USA; ^2^Department of Diagnostic Radiology, Mercy Catholic Medical Center, Mercy Fitzgerald Hospital, Darby, PA 19023, USA; ^3^Philadelphia College of Osteopathic Medicine, Philadelphia, PA 19131, USA

## Abstract

After presenting for a routine screening exam, and 57-year-old man was diagnosed with an incidentaloma—a primitive neuroectodermal tumor (PNET) of the thymus. A member of the Ewing sarcoma family of tumors, a PNET is typically regarded as a malignancy of childhood and adolescence, usually occurring in the central nervous system. In the case at hand, our patient had an extremely unusual presentation, given his age and tumor location. Initial presentation is the only predictor for long-term survival. Current treatment recommendations advocate complete surgical resection whenever possible, radiation therapy, and adjuvant versus neoadjuvant chemotherapy.

A 57-year-old man presented for routine EGD as followup for his Barrett's esophagus, and upon complaining of palpitations, was referred to the Emergency Department. An EKG revealed new-onset atrial fibrillation. On subsequent workup, a nuclear stress test was performed, but the study was deemed limited given the marked amount of radioactive uptake in the upper thorax. Suspicious for a mass lesion, a CT of the chest was performed. A heterogeneously enhancing anterior mediastinal mass was found, with differential diagnosis including thymic carcinoma, germ cell tumor, or lipoma. An ultrasound-guided biopsy was performed, which revealed a trabecular neoplasm highly suggestive of neuroendocrine tumor. PET tumor imaging showed the anterior mediastinal mass to have intense FDG activity, which in correlation with the pathology report, favored thymic carcinoma. There was no evidence of lymph node or distant metastases. The surgical service was consulted for tumor debulking, and a sternotomy with mediastinal exploration was planned. The tumor was very vascular. It extended into the right pleural space, and even deep into the left pleural cavity, going up to the thoracic inlet. The posteroinferior aspect of the tumor was overlying the great vessels. The tumor was infiltrating the innominate vein and left phrenic nerve. Due to tumor extent and infiltration into surrounding structures, extensive surgical resection or thymectomy was abandoned. Surgical pathology showed a malignant neuroendocrine tumor histologically and immunophenotypically consistent with a peripheral primitive neuroectodermal tumor.

Primitive neuroectodermal tumor (PNET), a member of the Ewing sarcoma family of tumors (ESFTs), is typically regarded as a malignancy of childhood and adolescence, usually occurring in the central nervous system. Although rare, so-called peripheral PNETs do exist, usually found in the deep soft tissues of the trunk and lower limbs. In the case at hand, our patient had an extremely unusual presentation, given his age, tumor location, and presentation (incidental after cardiology workup). A systematic review reveals the presented case to be only the second case of documented thymic PNET in the English literature [1]. 

Pathologically, the ESFTs are classically described as small round blue cell tumors of neural crest derivation with differentiation along a neuroendocrine lineage [2]. 

Pathology reports for our patient's tumor revealed the following immunochemistry: positive for CD99, cytokeratin, CD57, and NSE; negative for TTF-1, CK7/CK20, CD56, synaptophysin, chromogranin, CD117, PLAP, S100, CD30, AFP, and CD1a. Histology revealed a poorly differentiated trabecular neoplasm highly suggestive of a peripheral neuroectodermal tumor (Figure 1). 

Radiographically, PNET imaging is nonspecific, with use (of CT or MRI) aiding primarily in defining tumor scope, treatment strategy, and the evaluation of therapeutic outcomes [3]. The appearance of tumor heterogeneity on imaging represents areas of hemorrhage and necrosis, as was the case in our patient's large lesion (Figure 2) [4].

Initial presentation is the only predictor for long-term survival, with primary tumor-only presentation having a 5-year survival rate of 60%, compared to 33% for primary plus metastatic disease [2, 5]. Overall survival rates in adults are in line with pediatric study outcomes [6]. 

Current treatment recommendations advocate complete surgical resection whenever possible, radiation therapy, and adjuvant versus neoadjuvant chemotherapy. In the pediatric population, treatment for PNET typically lasts 6–9 months and consists of alternating courses of 2 chemotherapeutic regimens: (1) vincristine, doxorubicin, and cyclophosphamide and (2) ifosfamide and etoposide. Treatment is similar for adults [7]. 

Our patient was scheduled for concurrent chemoradiation with curative intent. Outcome was optimistic, and an uncomplicated treatment course was carried out.

##  Conflict of Interests

All authors confirm that there is no conflict of interests to declare.

##  Authorship

All authors had full access to the data and a role in writing the paper.

## Figures and Tables

**Figure 1:**
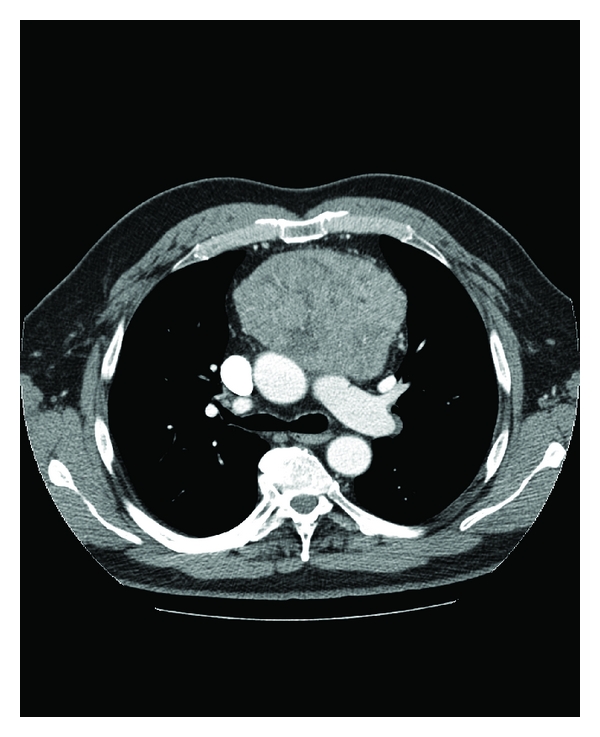
CT scan of the chest revealing a large heterogenous mass in the anterior mediastinum.

**Figure 2:**
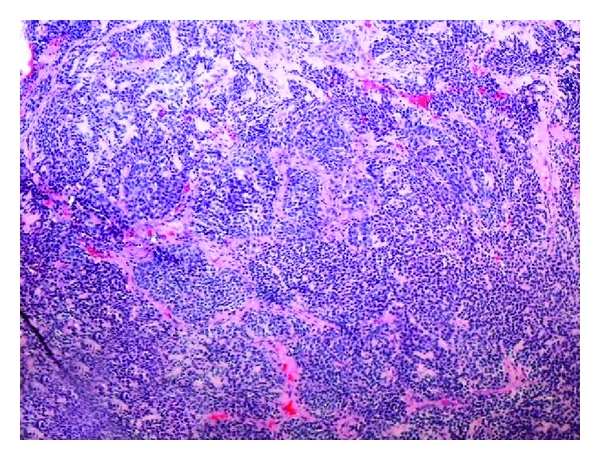
Pathology slide revealing a CD99+ poorly differentiated trabecular neoplasm consistent with a neuroendocrine tumor.
